# A critical review of chemical heterogeneity–driven microstructure control in steels

**DOI:** 10.1186/s42649-026-00139-5

**Published:** 2026-06-09

**Authors:** Seung Hyun Lee, Ji Hoon Kim

**Affiliations:** 1 School of Materials Science and Engineering, Pusan National University, 2, Busandaehak-Ro 6 Beon-Gil, GeumJeong-Gu, Busan, 46241 Republic of Korea; 2 Institute of Materials Technology, Pusan National University, 2, Busandaehak-Ro 6 Beon-Gil, GeumJeong-Gu, Busan, 46241 Republic of Korea

## Abstract

This paper reviews a recent microstructure control strategy for steels based on intentional chemical heterogeneity, referred to as “chemically heterogeneous steels” or “chemically patterned steels.” This approach introduces controlled chemical inhomogeneity, which has traditionally been avoided, in order to diversify microstructural design and enhance mechanical properties. The strategy involves a three-step process: (i) introducing compositional variations in a multiphase region, (ii) preserving these variations through rapid austenitization, and (iii) controlling microstructural evolution during subsequent cooling. Chemical heterogeneity is introduced via austenite reversion, carbide precipitation, or pearlitic microstructures, enabling precise control of concentration gradients and characteristic length scales. Building on this processing route, the review emphasizes two primary mechanisms for property improvement. First, localized elemental partitioning stabilizes a higher fraction of retained austenite with a broad stability spectrum, inducing a progressive transformation-induced plasticity (TRIP) effect that enhances ductility. Second, the resulting “chemical boundaries” act as physical barriers to dislocation motion and crack propagation, increasing strength and resistance to hydrogen embrittlement. Finally, the paper suggests extending this concept beyond Mn to other alloying elements such as Cr and Ni, as well as its potential application to titanium alloys and implementation in industrial-scale production lines.

## Introduction

The automotive, transportation, and defense industries increasingly demand advanced high-strength steels (AHSS) that exhibit both high strength and excellent ductility to achieve improved fuel efficiency, crash safety, and environmental sustainability (Aydin et al. 2013; Hance 2018). In particular, the development of third-generation AHSS has focused on achieving an excellent strength-ductility balance with low alloy designs combined and the stabilization of retained austenite (Bleck et al. 2017; Soleimani et al. 2020). By retaining metastable austenite at room temperature, these steels exploit the transformation-induced plasticity (TRIP) or twinning-induced plasticity (TWIP) effects during deformation.

For instance, medium-Mn steels processed through intercritical annealing (IA) stabilize austenite through the partitioning of Mn and C. However, the presence of soft ferrite in the resulting microstructure often leads to a decrease in yield strength and degraded surface quality due to Lüders banding (He et al. 2018; Wang et al. 2019, 2025). Second, quenching and partitioning (Q&P) and bainitic steels that processed by cooling to intermediate temperature after partitioning have higher yield strength than medium-Mn steels. However, only C can diffuse at these relatively low partitioning temperatures. Mn cannot diffuse and does not contribute to austenite stabilization (Xie et al. 2014; Zhang et al. 2022a). Furthermore, conventional Q&P or bainitic steels often require additional Si or Al to suppress carbide precipitation and stabilize austenite (Kwok & Dye 2023; Zhang et al. 2025). And it can make poor surface coatability and castability (Barbé et al. 2006; Zhang et al. 2022a). As a result, relying on homogeneous microstructural control makes it challenging to achieve high yield strength and maximize the austenite-stabilizing potential of Mn. (Ding et al. 2020; Kozłowska et al. 2023).

To overcome these challenges, the recently proposed chemical heterogeneity alloy strategy exploits controlled chemical heterogeneity through a different thermo-kinetic route, departing from conventional homogenization. Historically, the thermodynamic concept of utilizing intentional elemental partitioning to stabilize austenite has been proposed previously, such as in the quenching-lamellarizing-tempering (QLT) process for 5.5–9% Ni steels (Kim et al. 1983). However, the essential of this new approach lies in the successful preservation of previous Mn's chemical partitioning difference even after the entire microstructure goes through full austenitization process at high temperatures. By controlling these remained spatial concentration gradients and the resulting chemical boundaries to control subsequent phase-transformation behavior this approach overcomes the low yield strength of conventional medium-Mn steels and enables Mn partitioning unavailable in Q&P or bainitic steels to maximize austenite stability (Liu et al. 2020b; Kim et al. 2022a, 2024; Wang et al. 2024b; Zhang et al. 2025). Consequently, it achieves superior strength-ductility synergy and fracture resistance. Steels developed based on this concept are commonly referred to as ‘chemically heterogeneous steel’ or ‘chemically patterned steel’.

In this review, we comprehensively summarize the processing routes used to introduce and control chemical heterogeneity in multiphase steels. We explain the mechanisms of elemental distribution, microstructural evolution, and the resulting phase-transformation behaviors during cooling. Moreover, we discuss how chemical boundaries and enhanced austenite stability contribute to mechanical properties, and we highlight unexplored directions and potential industrial applications for chemically heterogeneous steels.

## Heat treatment process of chemically heterogeneous steel

In general, when heat treating conventional steels, a fully austenitized and chemically uniform microstructure is first obtained through austenitization, followed by cooling to room temperature along different heat-treatment paths to design various microstructures.

**Fig. 1:**
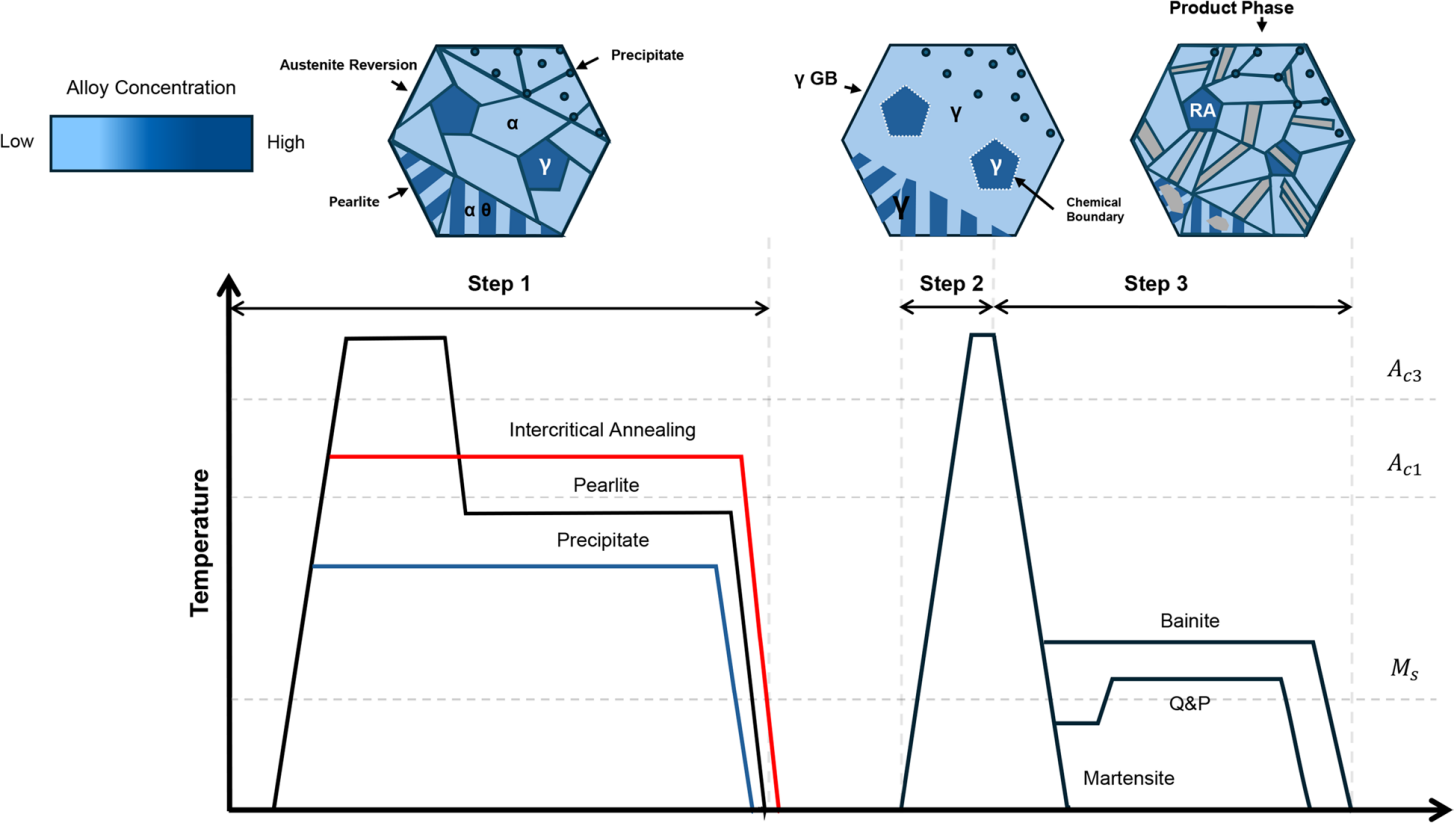
Schematic illustration of the three-step heat-treatment process for producing chemically heterogeneous steel. Step 1 introduces chemical heterogeneity in a multiphase region. Step 2 achieves full austenitization while preserving the chemical heterogeneity. Step 3 controls the transformation during cooling to obtain the desired microstructures, such as martensite or bainite

In contrast, chemically heterogeneous steels introduce compositional heterogeneity prior to austenitization by applying heat treatment within a multiphase region (such as austenite, ferrite, cementite, pearlite in steel). Accordingly, the processing route for chemically heterogeneous steels can be divided into three main heat-treatment steps (Fig. 1). The first step is the intentional introduction of chemical heterogeneity by annealing at multi-phase region (Step 1). The second step involves full austenitization while preserving the chemical heterogeneity introduced in the first step (Step 2). Because chemical heterogeneity formed in the first step must be retained during high-temperature austenitization, flash heating and short holding time are required in this stage. Finally, the third step involves applying various cooling and heat-treatment paths to the chemically heterogeneous austenite in order to generate distinct microstructures (Step. 3).

### Heat treatment step 1: introducing chemical heterogeneity

Steels are representative metallic materials that undergo high-temperature phase transformations, exhibiting a body-centered cubic (BCC) ferrite phase that is stable at low temperatures and a face-centered cubic (FCC) austenite phase that is stable at high temperatures. In addition, when C is added, a carbide with an orthorhombic crystal structure, namely cementite, can precipitate. Because these constituent phases possess different crystal structures, they inherently exhibit different chemical compositions. Moreover, as the equilibrium phase fractions vary with temperature, the solubility of alloying elements differs significantly among the phases.

Consequently, when heat treatment is conducted within a multiphase region where ferrite, austenite, and cementite coexist, alloy partitioning can be induced due to differences in solubility among the constituent phases. Typically, Mn and Ni act as austenite-stabilizing elements and preferentially partition into the FCC phase, whereas elements such as Si, Al, and Cr are ferrite stabilizers and tend to partition into the BCC phase. C and Mn can also partition into cementite, enabling the introduction of various chemically heterogeneous distributions.

To date, chemically heterogeneous steels have utilized austenite reverse transformation, carbide or cementite precipitation, and pearlite (a lamellar structure composed of ferrite and cementite) as effective means to introduce chemical heterogeneity.

#### Utilization of austenite reverse transformation

**Fig. 2:**
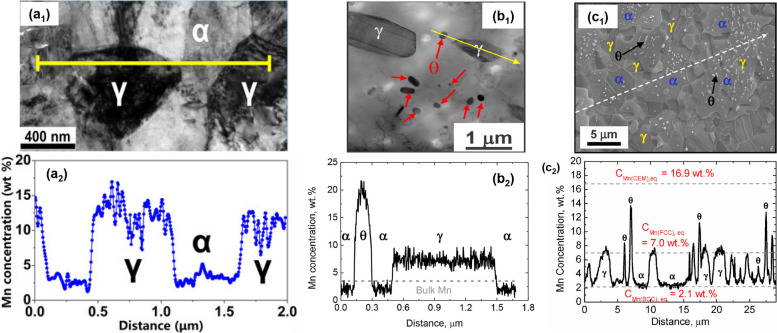
Representative microstructure and corresponding alloy element profile after austenite reversion. **a** TEM micrographs and Mn profiles of 0.18C – 8Mn steel after austenite reversion at 600 °C (Ding et al 2020), **b** TEM micrographs and Mn profiles of 0.18C – 3.5Mn – 0.1Si steel after austenite reversion and cementite precipitation (Kim et al. 2022a), **c** SEM micrographs and Mn profiles of 0.25C – 4Mn – 1.5Si steel after austenite reversion and cementite precipitation (Gu et al. 2022). All figures are reproduced with permission

The most widely studied approach for introducing chemical heterogeneity is utilizing austenite reversion in the two-phase (ferrite + austenite) region. During intercritical annealing (IA) between the Ac₁ and Ac₃ temperatures, austenite-stabilizing elements such as Mn partition into the reversed austenite. Consequently, Mn-enriched austenite and Mn-depleted ferrite are formed, inducing chemical heterogeneity (Liu et al. 2017, 2021; Ding et al. 2020; Kim et al. 2020, 2022a, 2024; Gu et al. 2022; Wang et al. 2024a, 2024b, 2025; Yan et al. 2024; Erişir et al. 2025). Representative microstructures and corresponding alloy concentration profiles are shown in Fig. 2.

Early studies primarily focused on the effect of intercritical annealing conditions on chemical heterogeneity. For instance, applying a relatively short treatment of 30 min to 2 h at 600–670 °C induces steep Mn concentration gradients (approximately 3–11 wt.%) within fine austenite grains smaller than 1 µm (Ding et al. 2020; Kim et al. 2020). Conversely, extending the holding time to 24 h allows for sufficient diffusion, resulting in relatively coarse austenite (~ 2.5 µm) with significant Mn heterogeneity.

Recently, beyond simple isothermal holding, advanced approaches have been attempted to further refine heterogeneity to the nanoscale by modifying the heating path or incorporating pre-treatments. Representative studies include forming fine Mn-enriched regions of ~ 0.4 µm by applying a slow continuous heating rate of 0.1 K s⁻^1^ instead of isothermal annealing to control phase transformation kinetics (Yan et al. 2024), or introducing a cryogenic pre-treatment (−196 °C) before IA to induce complex compositional fluctuations of Mn (~ 90 nm) and Ni (~ 75 nm) at a nanoscale below 100 nm (Wang et al. 2024a).

Furthermore, the control of austenite reversion is expanding beyond forming concentration gradients to control the morphology of austenite. The continuous IA treatment (e.g. 3 h + 2 h + 2 h) allows the formation of film-type and block-type austenites within Mn-rich regions at a time. Ultimately, this improves austenite stability, thereby increasing yield strength and suppressing Lüders band formation (Wang et al. 2025).

#### Utilization of carbide precipitation

**Fig. 3:**
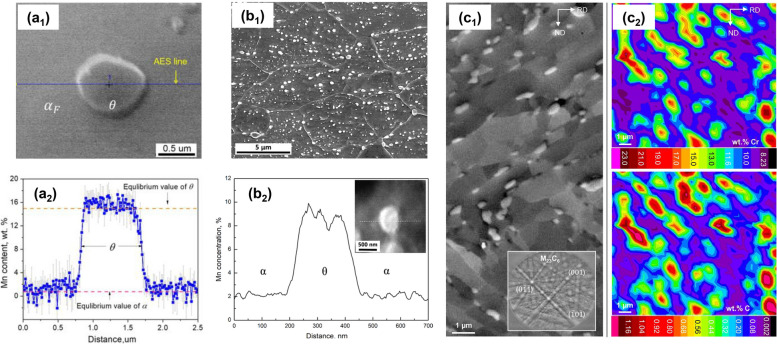
Representative microstructure and corresponding alloy element profile after carbide precipitation. **a** SEM micrographs and Mn profiles of 0.23C – 1.54Mn steel after cementite precipitation at 650 °C (Liu et al 2020a), **b** SEM micrographs and Mn profiles of 0.2C – 2Mn steel after cementite precipitation (Zhang et al. 2022a), **c** SEM micrographs and Cr and C distribution maps of 0.32C – 11.6Cr after carbide precipitation (Belde et al. 2015). All figures are reproduced with permission

Another effective strategy for introducing chemical heterogeneity is to promote carbide precipitation by conducting heat treatment at temperatures below Ac₁. During this process, supersaturated C in the matrix precipitates as carbides, while simultaneously, alloying elements such as Mn, Cr, and V partitions become enriched within these carbides. Although such carbides are smaller than that of austenite formed through reversion, it offers the advantage of inducing locally much steeper and higher concentration gradients of alloying elements (Belde et al. 2015, 2016; Liu et al. 2020a; Kim et al. 2021; Zhang et al. 2022a, 2025; Chai et al. 2023; Xu et al. 2023; Yu et al. 2025). Representative microstructures and the corresponding alloy enrichment phenomena are shown in Fig. 3.

This heterogeneity within the carbides is mainly governed by carbide coarsening and the partitioning of alloying elements, which depend on the heat treatment temperature and time. As the isothermal holding time increases, the morphological changes of the carbides and the internal enrichment of alloying elements become more pronounced. For example, as the annealing time at 580 °C increases from 0 to 10 h, cementite gradually spheroidizes from its initial lamellar structure and grows. During this morphological change, the internal Mn concentration of the carbide also increases from 3 wt.% up to 12 wt.% (Xu et al. 2023). When prolonged tempering is performed for 24 to 120 h at higher temperatures (600–650 °C), an Mn concentration gradient reaching 10–14 wt.% can be achieved within coarsened spherical cementite ranging from 250 nm to 1 µm in size (Liu et al. 2020a; Zhang et al. 2022a, 2025). In addition, applying a heat treatment just below the Ae₁ temperature to induce extremely fine, nanoscale (~ 53 nm) cementite with a moderate concentration gradient is also utilized for austenite stabilization (Kim et al. 2021).

Kinetic control methods aimed at maximizing the effects of heterogeneity by introducing multi-step annealing or morphology control are also being actively investigated. By first establishing a weak concentration gradient between austenite and martensite through intercritical annealing (760 °C) and then conducting a second heat treatment at 650 °C, cementite of ~ 115 nm in size with a maximized Mn concentration of ~ 17.5 wt.% in specific regions can be formed (Yu et al. 2025). Additionally, by controlling the initial morphology of the carbides (globular, banded, or lamellar), it is possible to implement a core–shell mechanism during subsequent processing, which consists of a residual carbide core, an austenite shell, and a martensitic matrix (Belde et al. 2016).

This carbide-utilizing strategy is applicable not only to Mn but also to strong carbide-forming elements such as Cr. In press-hardened steels (PHS) or high-C steels, carbides (M₇C₃, M₂₃C₆) formed through heat treatments in the 700–750 °C range can achieve internal Cr concentrations from 25 wt.% up to 40 wt.%, despite their fine size of 100–300 nm (Belde et al. 2015; Chai et al. 2023).

#### Utilization of pearlite

**Fig. 4:**
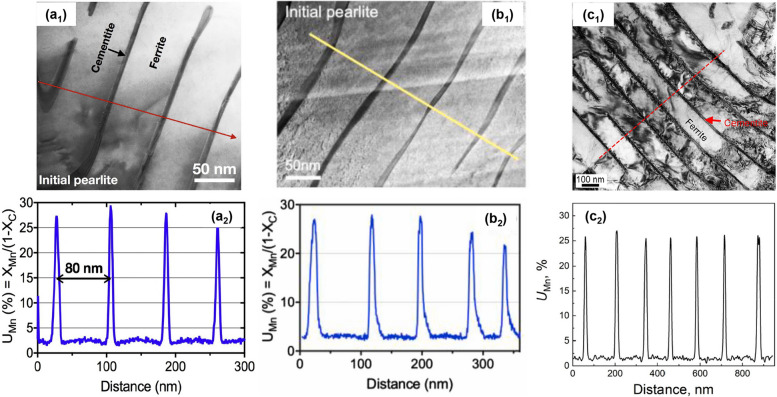
Representative microstructure and corresponding alloy element profile after pearlite transformation. **a** TEM micrographs and Mn profiles of 0.51C – 4.35Mn steel after pearlite transformation at 575 °C (Sun et al 2018), **b** TEM micrographs and Mn profiles of 0.45C – 4.58Mn steel after pearlite transformation at 575 °C (An et al. 2022), **c** TEM micrographs and Mn profiles of 0.39C – 3.69Mn steel after pearlite transformation at 570 °C (Yang et al. 2024a). All figures are reproduced with permission

Pearlite is a characteristic microstructure formed when austenite transforms into ferrite during cooling, resulting in C enrichment beyond the eutectoid composition and the subsequent formation of a lamellar structure consisting of ferrite and cementite. Similar to carbides, pearlite exhibits local enrichment of Mn and C; however, owing to its distinctive lamellar morphology, it enables the formation of unique microstructural features and has therefore been widely utilized as an effective means of introducing chemical heterogeneity (Sun et al. 2018; Li et al. 2019b; Yang et al. 2021, 2025; An et al. 2022; Zhang et al. 2022b; Gao et al. 2023; Abe et al. 2024; Liu et al. 2024a, 2024b; Ren et al. 2024; Xiong et al. 2024; Yang et al. 2024a, 2024b; Fan et al. 2025; Kaboli et al. 2026). Representative microstructures and corresponding alloy enrichment are shown in Fig. 4.

The characteristics of pearlite-induced heterogeneity—specifically, the lamellar spacing and the concentration gradient—are primarily governed by isothermal holding conditions (temperature and time) and the initial C content. For instance, applying isothermal holding at 500–650 °C for 1 to 36 h results in various lamellar spacings ranging from ~ 40 nm to ~ 150 nm, depending on the heat treatment parameters and alloy composition. Consequently, the Mn concentration gradient can also be controlled from ~ 7 wt.% up to ~ 26 wt.% (An et al. 2022; Gao et al. 2023; Ren et al. 2024). Furthermore, the scale of the microstructure formed during processing has a decisive impact on the degree of elemental partitioning. While relatively large cementite particles (> 500 nm) can accumulate high Mn levels of up to ~ 24 wt.%, finer pearlitic lamellar structures (< 300 nm) exhibit a more moderate enrichment of approximately 10 wt.% (Kaboli et al. 2026).

Spheroidization serves as another mechanism for tailoring chemical heterogeneity. By combining long-term heat treatments with mechanical deformation such as cold rolling, lamellar pearlite transforms into spheroidized cementite. This process alters the characteristic size of the pearlite (e.g. coarsening from ~ 0.16 μm to ~ 0.4 μm) while maintaining a strong Mn concentration gradient of 16–18 wt.% (Abe et al. 2024; Yang et al. 2024b). Strategically controlling the initial pearlite morphology plays a critical role in dictating the growth mode and final morphology of austenite during the subsequent austenitization process.

#### Comparison of chemical heterogeneity characteristics introduced by different constituent phases

**Fig. 5:**
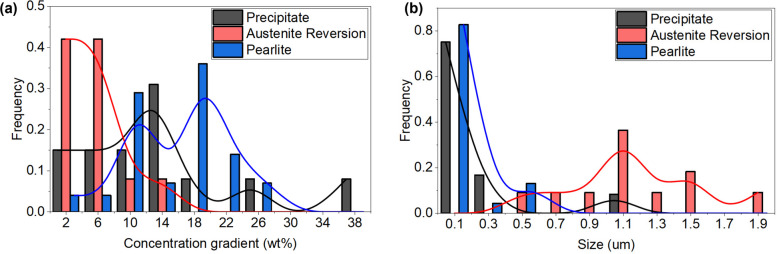
Chemical heterogeneity characteristics (concentration gradient and size) formed after introducing chemical heterogeneity, depending on the type of alloy-enriched constituents. **a** concentration gradient and (**b**) size distribution

Figure 5 illustrates the distribution of heterogeneity characteristics —namely the concentration gradient and characteristic length scale— introduced by utilizing different constituent phases for chemical heterogeneity. The concentration gradient was calculated after the Step 1 heat treatment (multiphase heat treatment for heterogeneity introduction) as the difference between the maximum alloy concentration in the enriched region and the minimum concentration in the depleted region, based on EDS or EPMA data, and then averaged. The characteristic length scale of heterogeneity was defined as the average distance between the alloy-enriched and alloy-depleted regions.

Figure 5a summarizes the concentration gradients reported in previous studies on chemically heterogeneous steels. As shown, steels utilizing austenite reversion generally exhibit concentration gradients below ~ 14 wt.%, whereas those employing carbides tend to show relatively higher concentration gradients over 18 wt.%. Pearlite-based approaches also display high concentration gradients, as they inherently involve cementite. Figure 5b presents the characteristic length scales of chemical heterogeneity. In the case of austenite reversion, the heterogeneity size is typically on the order of ~ 1 µm, whereas steels utilizing carbides and pearlite generally exhibit submicrometer length scales.

As demonstrated, the characteristics of chemical heterogeneity can be effectively controlled by selecting the constituent phases (austenite, carbide, or pearlite) used for heterogeneity introduction during the Step 1 heat treatment. These heterogeneity characteristics play a critical role in determining the processing conditions (austenitizing temperature and holding time) for the subsequent Step 2 single-phase high-temperature heat treatment, during which partial homogenization inevitably occurs, as well as the final microstructural morphology, since the driving force for phase transformation differs depending on the local chemical composition.

### Heat treatment step 2: austenitization while maintaining chemical heterogeneity

In chemically homogeneous steels, it is well established that the austenite grain size can be controlled by adjusting the austenitizing temperature and holding time. In general, higher temperatures and longer holding times lead to grain coarsening of austenite, and the austenite grain size strongly influences grain refinement in the final microstructures (ferrite, bainite, and martensite), thereby serving as an effective means of controlling mechanical properties. In addition, the austenite grain size affects the density of grain boundaries, which act as nucleation sites during cooling, and consequently influences the distribution of martensite within the microstructure (Prawoto et al. 2012; Karthikeyan et al. 2017; Singh et al. 2018; Xu et al. 2021; Kim et al. 2022b; Zhang et al. 2022c; Li et al. 2024).

**Fig. 6:**
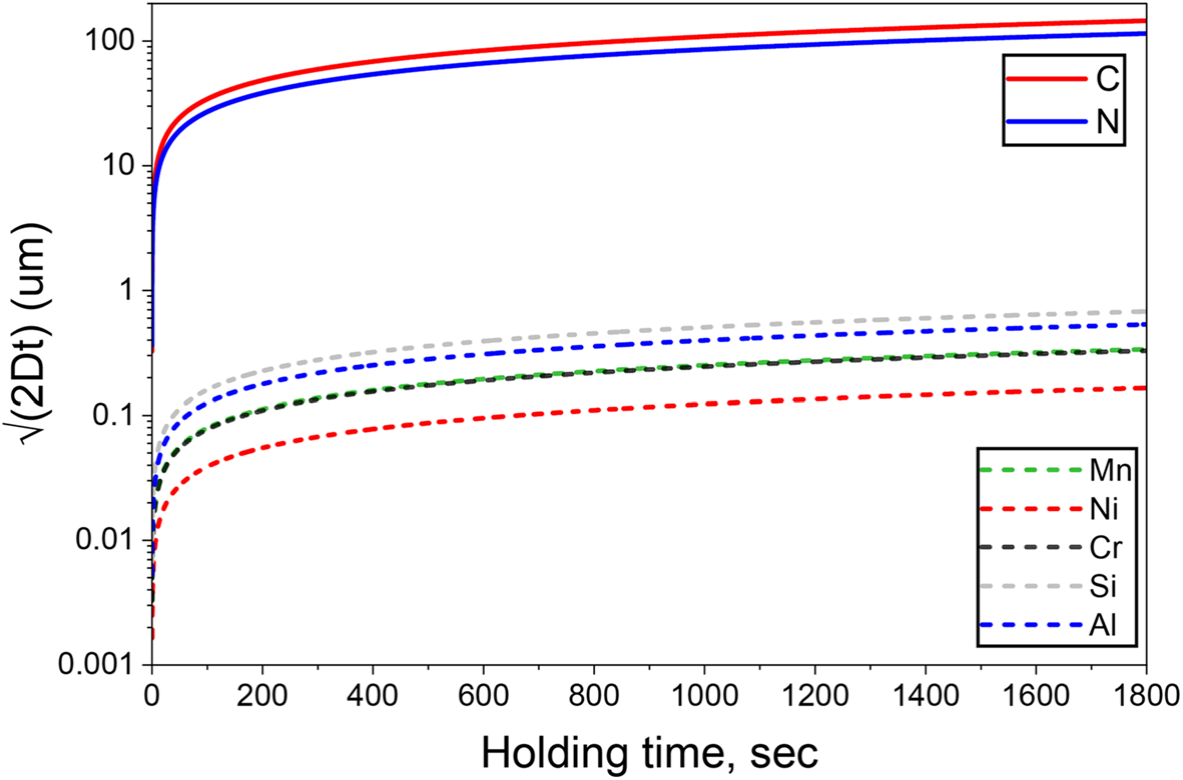
Calculated diffusion distance in FCC depending on holding time after annealing at 900 °C

In chemically heterogeneous steels, austenitization conditions similarly affect the austenite grain size, as in chemically homogeneous steels. However, a key additional factor is the homogenization that occurs during high temperature austenitization. Chemical heterogeneity introduced during the Step 1 heat treatment can be diminished or eliminated by diffusion during subsequent austenitization. Figure 6 presents calculated diffusion distances of alloying elements (C, N, Mn, Ni, Cr, Si, and Al) commonly used in advanced high-strength steels as a function of holding time at 900 °C, which corresponds to the conventional full austenitization temperature of steels. The diffusion distance was calculated the following equation considering the diffusion coefficient and activation energy of each alloying element in the FCC (austenite) phase.

$$\mathrm{x}=\sqrt{2\mathrm{D}\mathrm{t}}$$

$$D={D}_{0}\cdot\mathrm{exp}\left(\frac{-{Q}_{d}}{RT}\right)$$

$$\begin{aligned}\mathrm{t}&=\mathrm{holding}\;\mathrm{time}\;\left(\mathrm{s}\right)\\{D}&=\mathrm{diffusion}\;\mathrm{coefficient}\left(m^{2}/s\right)\;\\{D}_{0}&=\mathrm{pre-exponetial}\left(m^{2}/s\right)\end{aligned}$$

$$\begin{aligned}\mathrm{Q}_{d}&=\mathrm{activation}\;\mathrm{energy} \left(\mathrm{J/mol}\;\mathrm{or}\;\mathrm{eV}/\mathrm{atom}\right)\\{R}&=\mathrm{gas}\; \mathrm{constant} \left(8.314\mathrm{J}/\mathrm{mol}\cdot\mathrm{K}\right)\end{aligned}$$

$$\mathrm{T}=\mathrm{absolute}\;\mathrm{temperature} \left(\mathrm{K}\right)$$

Parameters for the calculation are adopted from (Oikawa 1982), (Bergner & Khaddour 1993), (Fridberg & Hillert 1970).

The diffusion distances of substitutional alloying elements (Mn, Ni, Cr, Si, and Al) are on the submicrometer scale, whereas those of interstitial elements (C and N) reach the micrometer scale. This indicates that if chemical heterogeneity with a micrometer-scale length scale is introduced during the Step 1 heat treatment, it can be retained even after full austenitization at high temperatures. In addition, the degree of chemical heterogeneity can be controlled by adjusting the austenitization temperature and holding time. Therefore, a critical aspect of austenitization in chemically heterogeneous steels is the careful selection of austenitization temperature and holding time to achieve the desired level of chemical heterogeneity for controlling microstructural evolution during cooling.

Based on this understanding, previous studies on the influence of austenitization processing parameters (heating rate, temperature, and holding time) on the microstructure of chemically heterogeneous steels are summarized below.

#### Effect of heating rate

The heating rate determines the mode of phase transformation during the transition from a multiphase microstructure formed after the Step 1 heat treatment to single-phase austenite. Specifically, it governs whether austenitization proceeds via interface migration accompanied by diffusion of substitutional alloying elements, corresponding to the partitioning local equilibrium (PLE) mode, or via interface migration involving only the diffusion of interstitial elements while suppressing substitutional diffusion, corresponding to the negligible partitioning local equilibrium (NPLE) mode.

In chemically heterogeneous steels, preservation of the chemical heterogeneity introduced during the Step 1 heat treatment is essential; therefore, austenitization via the NPLE mode is preferred. Such transformation behavior is generally achieved under rapid heating conditions, typically on the order of several tens to several hundreds of °C s⁻^1^. Consequently, numerous studies have focused on the effect of heating rate during the Step 2 austenitization heat treatment (Sun et al. 2018; Li et al. 2019b; Ding et al. 2020; Liu et al. 2020a; Gu et al. 2022; Ren et al. 2024; Wang et al. 2024b; Yan et al. 2024; Yang et al. 2024a). Because the preservation of chemical heterogeneity and the transition of austenite growth modes are controlled by heating rate, DICTRA simulation are frequently applied to predict growth mode during austenitization. These simulations allow for the quantitative prediction of element diffusion behavior across moving phase interfaces under various heating rates. Furthermore, thermodynamic-kinetic models are utilized to determine the exact transition between the PLE and NPLE modes by evaluating the competition between the driving force for interface migration and the limited diffusivity of substitutional elements. These models provide a theoretical basis for optimizing flash heating parameters.

**Fig. 7:**
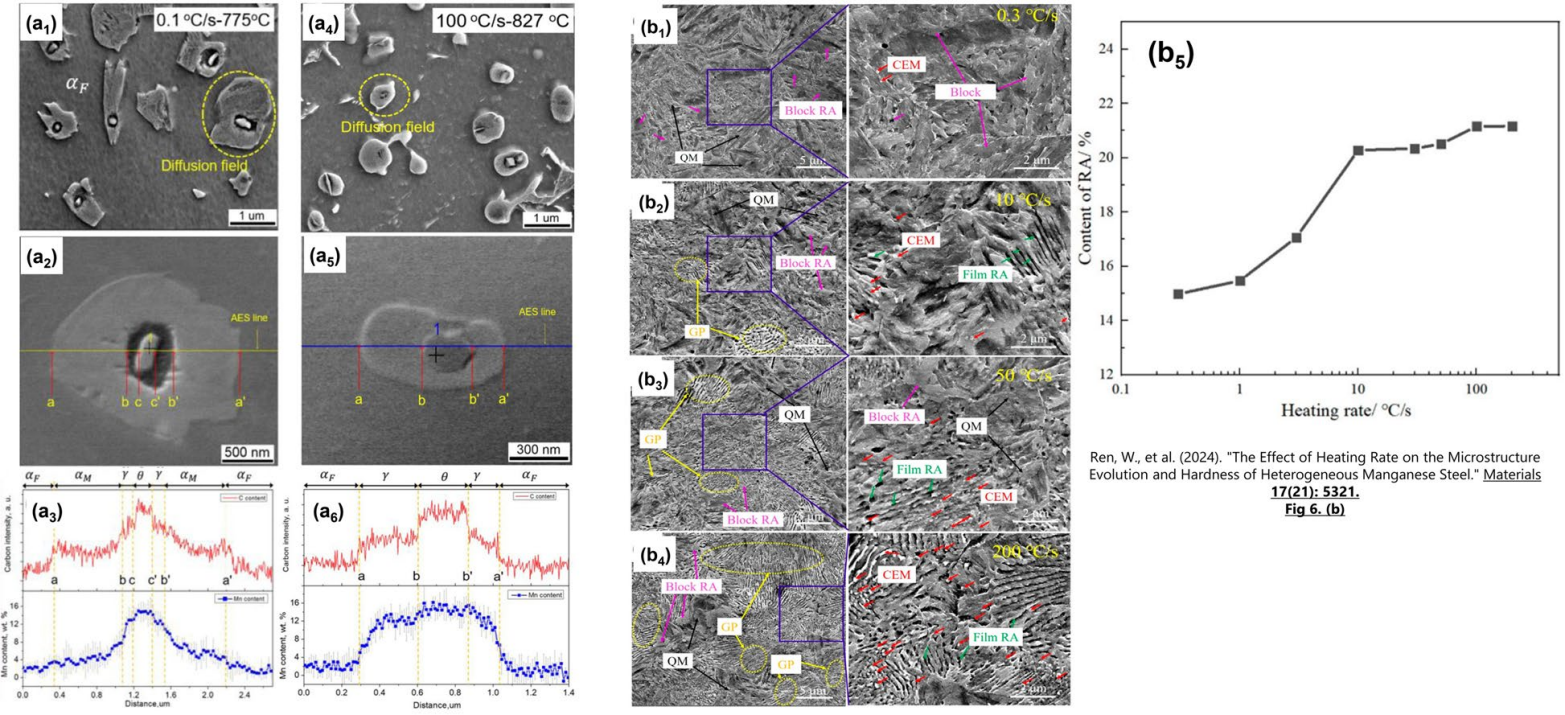
Influence of heating rate on the microstructure evolution of chemically heterogeneous steels. **a** Microstructures and corresponding Mn concentration profiles after austenitization of an initial cementite-containing microstructure using a slow heating rate of 0.1 °C/s (a_1_–a_3_) and a rapid heating rate of 100 °C/s (a_4_–a_6_) (Liu et al. 2020a). **b** Microstructures obtained after austenitization of an initial pearlitic microstructure at heating rates ranging from 0.3 °C/s to 200 °C/s (b_1_–b_4_), and the corresponding evolution of austenite fraction as a function of heating rate (Ren et al. 2024). All figures are reproduced with permission

The critical heating rate required to successfully preserve chemical heterogeneity depends on the characteristics of the initial microstructure. For microstructures containing Mn-enriched cementite or austenite, rapid heating (typically range from 30 °C s⁻^1^ to 100 °C s⁻^1^) is generally required to skip PLE-dominated growth. Under these rapid heating conditions, ferrite and cementite dissolve simultaneously, and the high Mn concentration originating from the initial phases is preserved, remaining heterogeneously distributed within the newly formed austenite (Liu et al. 2020a; Gu et al. 2022) (Fig. 7a).

Furthermore, the heating rate directly influences the microstructural evolution from specific initial structures like pearlite. S. Li et al. demonstrated that during the pearlite-to-austenite transformation, the transition between slow PLE-dominated growth and rapid NPLE-dominated growth occurs within a narrow temperature range, known as the PLE/NPLE transition temperature (PNTT). If the heating rate is sufficiently high to surpass this transition temperature, unique microstructures such as "ghost pearlite" are formed, which are characterized by residual Mn enrichment inherited directly from the prior pearlitic lamellar structure (Li et al. 2019b). Therefore, optimizing the heating rate is essential to maximize the stability of the final microstructure.

Additionally, W. Ren et al. reported that increasing the heating rate to an appropriate level (approximately 10 °C s⁻^1^) effectively preserves heterogeneity, significantly increasing the fraction of stable retained austenite. However, extreme heating rates beyond a certain threshold may not provide any additional benefits (Ren et al. 2024) (Fig. 7b). Although rapid heating is generally established as the standard for preserving chemical patterns, recent studies have shown that sufficient Mn chemical heterogeneity can also be maintained under relatively slow heating conditions (e.g. 0.1 °C s⁻^1^), depending on the specific alloy design and initial microstructural conditions (Yan et al. 2024; Yang et al. 2024a).

#### Effect of austenitization temperature and holding time

**Fig. 8:**
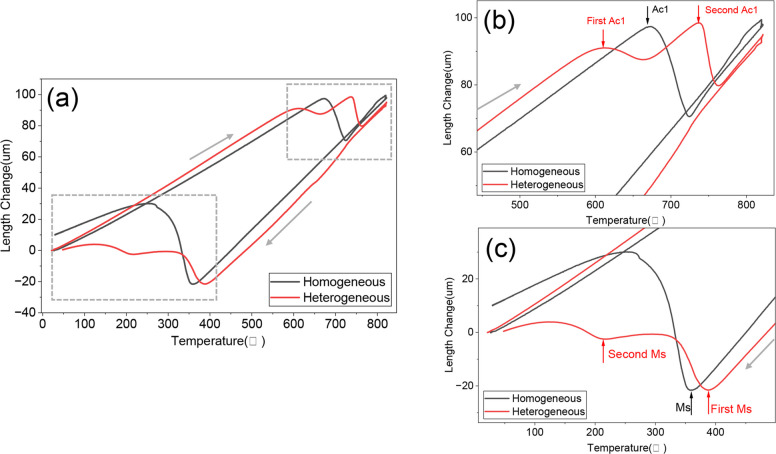
**a** Dilatometer curves of chemically homogeneous steel (black) and heterogeneous steel (red). **b** Magnified figure during heating for full austenitization and austenite transformations were identified with volume contraction. **c** Magnified figure during heating and martensitic transformations were observed by volume expansion. (author’s private data)

In chemically homogeneous steels, the chemical composition is uniform, and therefore a single Ac_3_ temperature exists at which complete austenitization occurs. In contrast, in chemically heterogeneous steels, the local chemical composition varies spatially, resulting in different Ac_1_ temperatures depending on the local composition. As shown in Fig. 8b, during heating, the ferrite (BCC)–to–austenite (FCC) transformation can be identified by the associated volume contraction. While this transformation typically occurs in a single step for homogeneous steels, chemically heterogeneous steels commonly exhibit two-step or multi-step austenitization during heating.

Moreover, even within the same specimen, the temperature required for complete austenitization in chemically heterogeneous steels is generally higher than that of chemically homogeneous steels. Therefore, this effect must be carefully considered when selecting the full austenitization temperature for chemically heterogeneous steels.

**Fig. 9:**
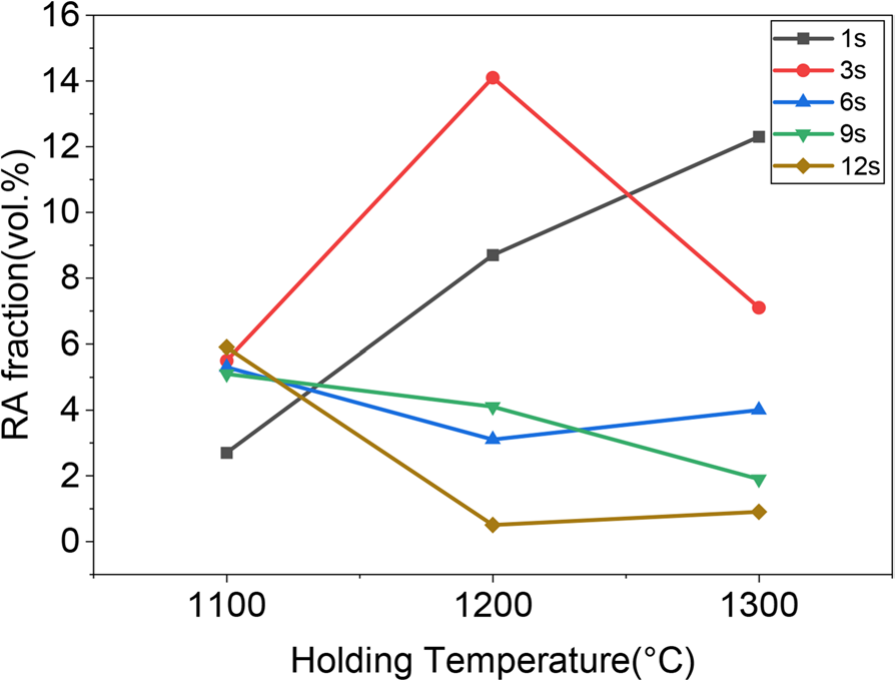
Effect of austenitizing (holding) temperature and time on retained austenite fraction (Belde et al. 2015). All figures are reproduced with permission

**Fig. 10:**
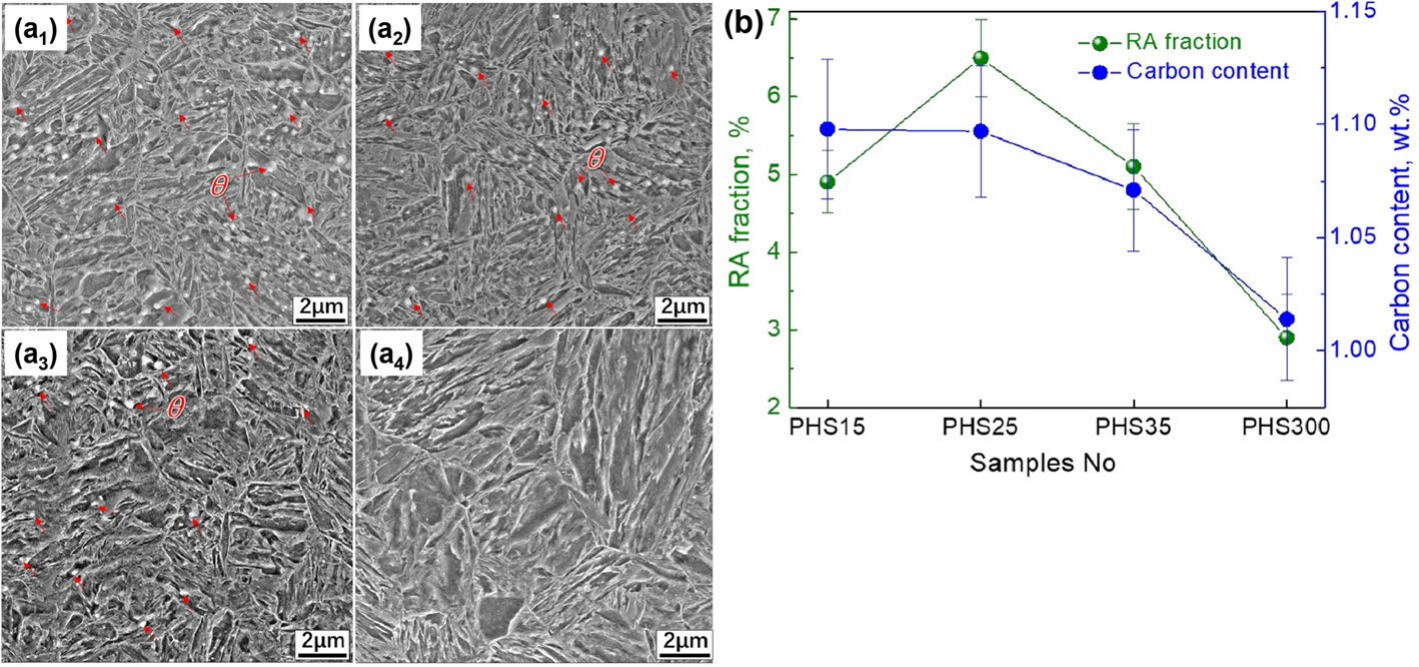
Effects of austenitizing (holding) time on chemical heterogeneity introduced by cementite in a 0.37C–1.6Si–1.5Mn–0.6Cr–0.4Ni–0.056Nb steel. (**a**1) 15 s holding, (**a**2) 25 s holding, (**a**3) 35 s holding, and (**a**4) 300 s holding. (**b**) Evolution of retained austenite fraction and C concentration as a function of holding time from 15 to 300 s (after Ding et al. 2025). All figures are reproduced with permission

Therefore, it is important to control the austenitizing temperature and holding time during the second-step heat treatment. These parameters should be designed to provide sufficient austenitizing temperature and time to make full austenite, while preventing from elements homogenization. For example, applying a short holding time of 1 s at 1200 °C would lead to incomplete dissolution of the carbide, resulting in a lower residual austenite (RA) ratio. However, increasing the temperature to 1300 °C or extending the holding time to more than 3 s would lead to homogenization due to diffusion of alloying elements, ultimately reducing the RA ratio. As a result, the highest RA ratio was achieved with a holding time of 3 s (Belde et al. 2015) under austenitizing conditions at 1200 °C (Fig. 9). Similarly, microstructures with Mn/Cr-enriched precipitates exhibit a maximum RA fraction at an optimal holding time of 25 s at 910 °C, followed by a continuous decrease up to 300 s (Ding et al. 2025) (Fig. 10). In other studies, initial pearlitic microstructures reach a peak RA fraction of ~ 19% when held at 750 °C for 20 s, but this value drops sharply to ~ 9.3% as the holding time is prolonged (Yang et al. 2021). Therefore, controlling these narrow time–temperature processing relationships plays a critical role in optimizing the microstructural control of chemically heterogeneous steels.

### Heat treatment step 3: application of various heat treatment path to chemically heterogeneous steel

Through the Steps 1 and Step 2 heat treatments, a chemically heterogeneous single-phase austenitic microstructure is first established, after which various heat-treatment paths are applied to produce different advanced high-strength steel (AHSS) microstructures. Because chemical composition directly governs the driving force for phase transformations, chemically heterogeneous austenite gives rise to locally varying transformation driving forces, leading to phase-transformation behaviors that differ from those observed in chemically homogeneous steels. Therefore, microstructures distinct from those of conventional steels are formed, accompanied by significantly different mechanical properties.

To date, the most extensively investigated steel types based on this concept include martensitic steels obtained by rapid cooling, bainitic steels produced via isothermal heat treatment above the martensite start temperature, TRIP (transformation-induced plasticity) steels formed through continuous cooling, and quenching and partitioning (Q&P) process, which are martensite-based TRIP steels. In the following sections, previous studies applying the concept of chemically heterogeneous steels to these representative steel classes are summarized.

#### Application to martensitic steel

Martensite is an essential phase for achieving high strength in steels and is generally obtained by rapid cooling from a single-phase austenitic state to room temperature. The martensite start temperature (M_s_) is well known to be a function of the chemical composition. In chemically homogeneous steels, martensitic transformation typically occurs in a single step. In contrast, in chemically heterogeneous steels, the M_s_ temperature varies locally according to compositional heterogeneity, leading to multi-step martensitic transformation behavior (Fig. 8c).

Although martensitic steels exhibit exceptionally high strength, their application is often limited by low ductility, with total elongation typically below 10%. However, recent studies have demonstrated that the application of the chemically heterogeneous steel concept can simultaneously enhance both strength and ductility in martensitic steels. As a result, considerable research efforts have been devoted to martensitic steel designed based on chemical heterogeneity (Liu et al. 2020b; Kim et al. 2021, 2022a; Liu et al. 2021; Zhang et al. 2025; Kaboli et al. 2026).

The enhancement of mechanical properties in chemically heterogeneous martensitic steels is mainly caused by two microstructural mechanisms: the stabilization of retained austenite and the crystallographic control of the martensite matrix itself.

First, the localized enrichment of alloying elements, originating from initial microstructures, lowers the Ms temperature in specific regions. This enables the retention of stable, nanoscale austenite within the hard martensite matrix. Consequently, it allows for improved ductility through a sustained TRIP effect, without sacrificing the inherent strength of the martensite (Kim et al. 2021, 2022a).

**Fig. 11:**
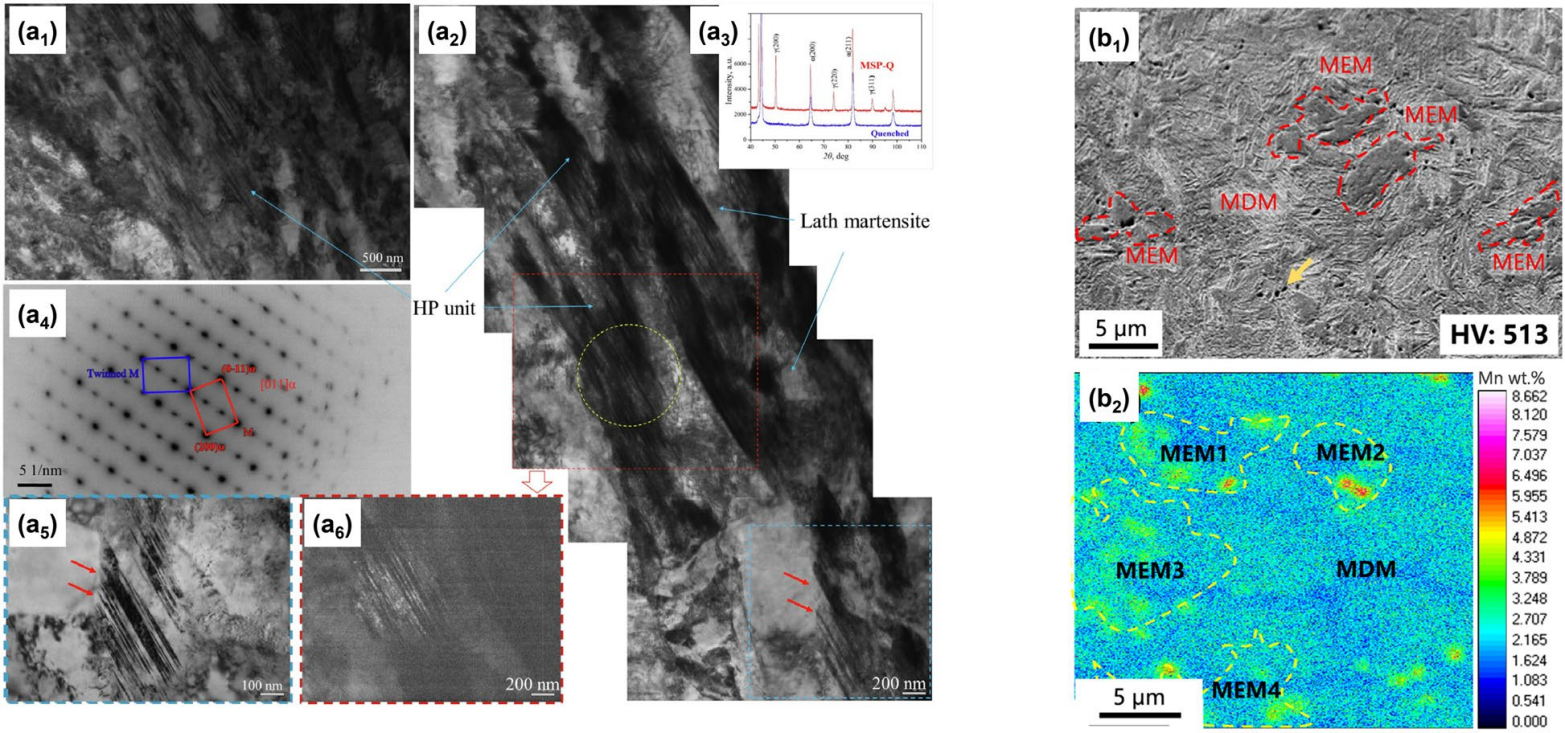
Microstructures of chemically heterogeneous martensitic steels. **a** Formation of nano-twinned martensite in Mn-enriched regions (Liu et al. 2021). **b** SEM micrographs and corresponding Mn distribution maps of a chemically heterogeneous martensitic steel, where MEM denotes Mn-enriched martensite and MDM denotes Mn-depleted martensite (Kaboli et al. 2026). All figures are reproduced with permission

Second, the chemical heterogeneity of elements such as Mn controls the substructure of the newly formed martensite. For instance, local variations in Mn concentration leads to the formation of typical lath martensite in Mn-depleted regions, while generating nano-twinned martensite in Mn-enriched regions (Fig. 11a) (Liu et al. 2021). Furthermore, this approach can be utilized to design a composite martensitic structure even under conditions where the retained austenite fraction is limited to less than 2%. By partitioning the microstructure into Mn-enriched, high-strength martensite (MEM) and Mn-depleted, low-strength martensite (MDM) regions, a synergistic effect between the hard and soft domains enhances mechanical properties (Fig. 11b). This demonstrates that the ductility enhancement mechanism in these steels relies not only on the TRIP effect but also on the intrinsic properties of the heterogeneous martensitic matrix and its strain partitioning capability (Kaboli et al. 2026).

#### Application to bainitic steel

Bainite is also one of the constituent phases frequently employed in high-strength steels. Bainitic transformation is achieved by isothermal heat treatment at temperatures above M_s_. Because the transformation occurs above M_s_, C partitions from bainitic ferrite into the untransformed FCC phase during transformation, thereby stabilizing austenite. As a result, bainitic steels can achieve a favorable combination of high strength and ductility through the TRIP effect of retained austenite.

In chemically heterogeneous steels, the driving force for bainitic transformation varies locally depending on the chemical composition, which in turn affects transformation kinetics, leading to either acceleration or retardation of bainitic transformation. In chemically homogeneous steels, austenite is stabilized primarily through C partitioning during bainitic transformation. In contrast, in chemically heterogeneous steels, austenite is stabilized by the combined effects of pre-partitioned Mn introduced prior to transformation and C partitioning during bainitic transformation, making chemical heterogeneity particularly effective in enhancing retained austenite stability. (Gao et al. 2023; Kim et al. 2024; Liu et al. 2024b; Yan et al. 2024; Fan et al. 2025).

**Fig. 12:**
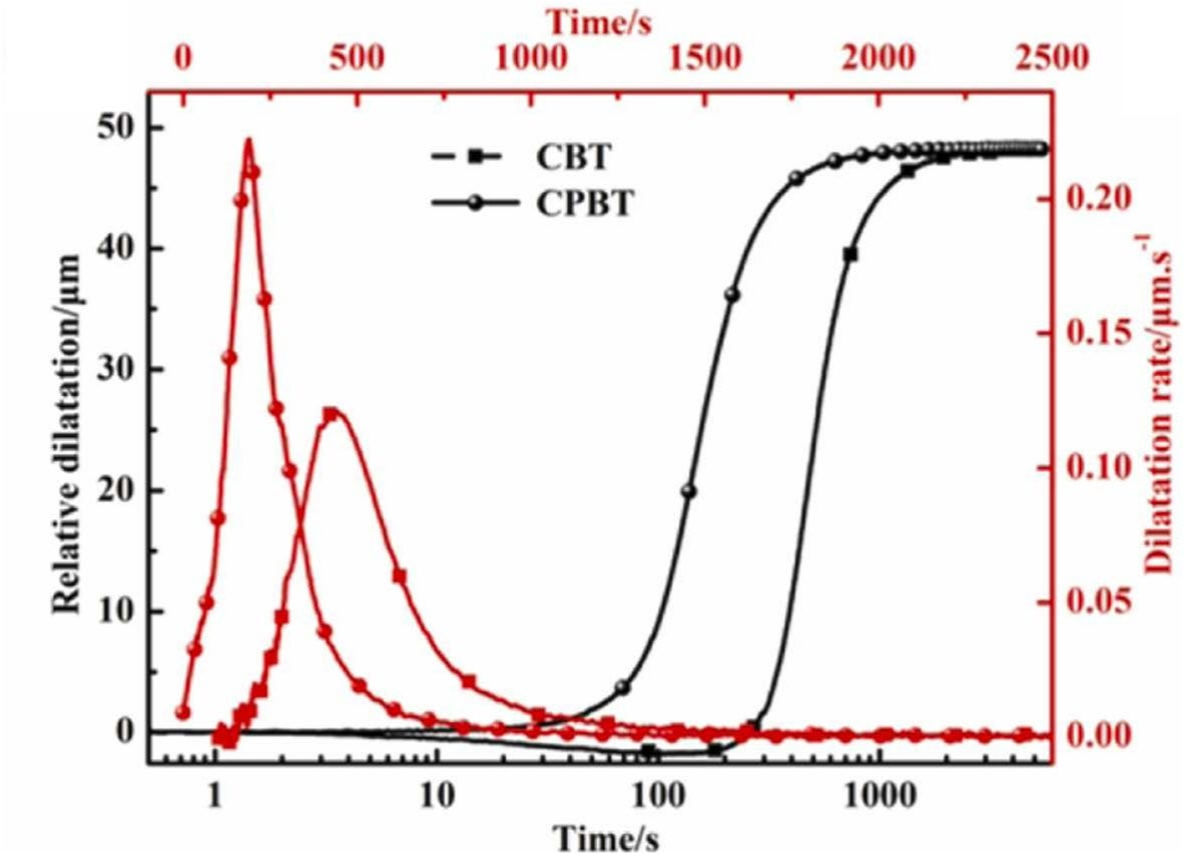
Comparison of bainitic transformation kinetics between chemically heterogeneous (CPBT) and chemically homogeneous (CBT) steels. The CPBT exhibits an accelerated bainitic transformation, primarily attributed to the presence of chemical heterogeneity (Guo et al. 2026). All figures are reproduced with permission

Bainite nucleation is preferentially promoted in Mn-lean regions. This nucleation accelerates the bainitic transformation kinetics, shortening both the incubation period and the transformation time (Fig. 12). Furthermore, this chemical patterning induces extensive intragranular nucleation within the Mn-lean domains. This transformation behavior contrasts with conventional chemically homogeneous steels, where bainite nucleation primarily occurs at prior austenite grain boundaries (Gao et al. 2023; Kim et al. 2024; Guo et al. 2026).

However, in Mn-enriched regions, untransformed austenite is preferentially stabilized by a dual stabilization effect (pre-partitioned Mn and partitioned C). This stabilization mechanism provides a unique pathway to control factors such as the size, morphology, and distribution of the retained austenite (Liu et al. 2024a; Yan et al. 2024).

#### Application to TRIP (Transformation Induced Plasticity) steel

As discussed earlier, martensitic steels inherently contain a very limited amount of retained austenite; however, the introduction of chemical heterogeneity has enabled effective stabilization of austenite, leading to improved mechanical properties. This demonstrates that chemical heterogeneity can serve as an effective strategy for stabilizing austenite. Accordingly, introducing chemical heterogeneity into TRIP steels—whose mechanical properties are intrinsically enhanced through retained austenite stabilization—can be expected to further improve austenite stability and mechanical performance.

Various heat-treatment routes have been developed to stabilize austenite in TRIP steels. Among them, this section summarizes previous studies in which the chemically heterogeneous steel concept was applied to TRIP steels produced via continuous cooling and quenching and partitioning (Q&P) processes (Liu et al. 2017; Gu et al. 2022; Zhang et al. 2022a, 2022b, 2024; Xiong et al. 2024; Yang et al. 2024a, 2025).

**Fig. 13:**
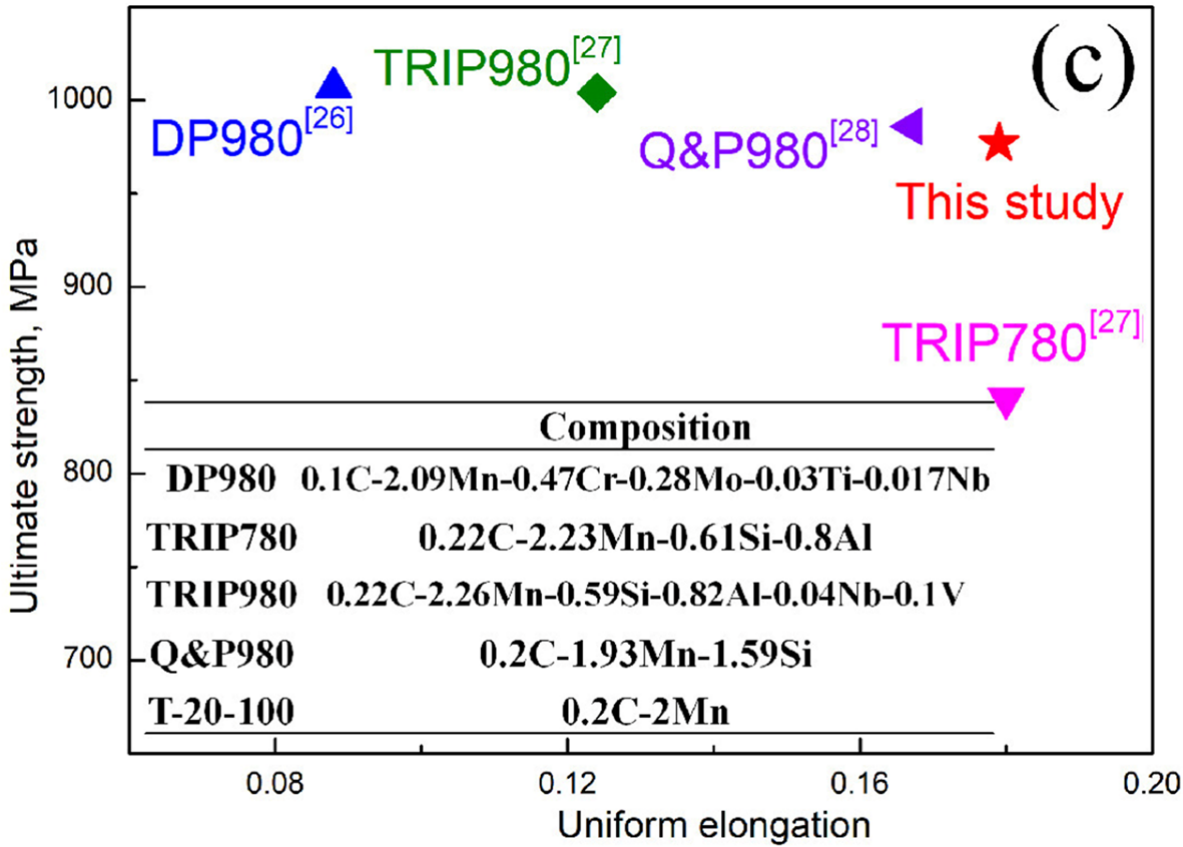
Strength and total elongation balance of conventional chemically homogeneous steel and chemically heterogeneous steel (red star symbol) (Zhang et al. 2022a). All figures are reproduced with permission

First, in the continuous cooling process, chemical heterogeneity promotes C partitioning and optimizes alloy design. In conventional chemically homogeneous TRIP steels, the substantial addition of elements such as Si or Al is essential to suppress carbide precipitation and ensure C partitioning from ferrite to austenite. However, introducing a chemical pattern (e.g. heterogeneity via initial cementite dissolution) thermodynamically favors austenite stabilization, enabling effective C partitioning even in Si- and Al-free alloy designs. As shown in Fig. 13, exploiting this heterogeneity not only secures excellent tensile properties but also provides a solution to severe industrial processing issues associated with high Si/Al contents, such as nozzle clogging during continuous casting and liquid metal embrittlement (LME) (Zhang et al. 2022a, 2022b; Xiong et al. 2024).

Second, in the Q&P process, chemical heterogeneity induces a multi-step martensitic transformation that simplifies the heat treatment process. The conventional Q&P process demands precise temperature controls specifically, an interrupted quenching step between the M_s_ and M_f_ temperatures—to allow C partitioning from the primary martensite into the untransformed austenite. However, the heterogeneous distribution of elements like Mn changes the local M_s_ and M_f_ temperatures. Crucially, in the enriched domains, the local M_f_ temperature decreases well below room temperature. Consequently, the interrupted quenching step is not necessary. Simply cooling the steel to room temperature prevents complete martensitic transformation and spontaneously induces a "Q&P-like" stabilization mechanism. This approach not only yields stable retained austenite and outstanding mechanical properties but also enhances the robustness and practicality of Q&P heat treatments for industrial-scale applications (Liu et al. 2017; Gu et al. 2022; Yang et al. 2024a, 2025; Zhang et al. 2024).

## Mechanical properties and their enhancing mechanisms

In the preceding sections, the processing routes for producing chemically heterogeneous steels and the effects of various processing parameters on microstructural evolution were reviewed. In this section, we focus on the mechanical properties of chemically heterogeneous steels, especially (i) strength, (ii) ductility, and (iii) performance under extreme environments.

### Enhancement of strength

**Fig. 14:**
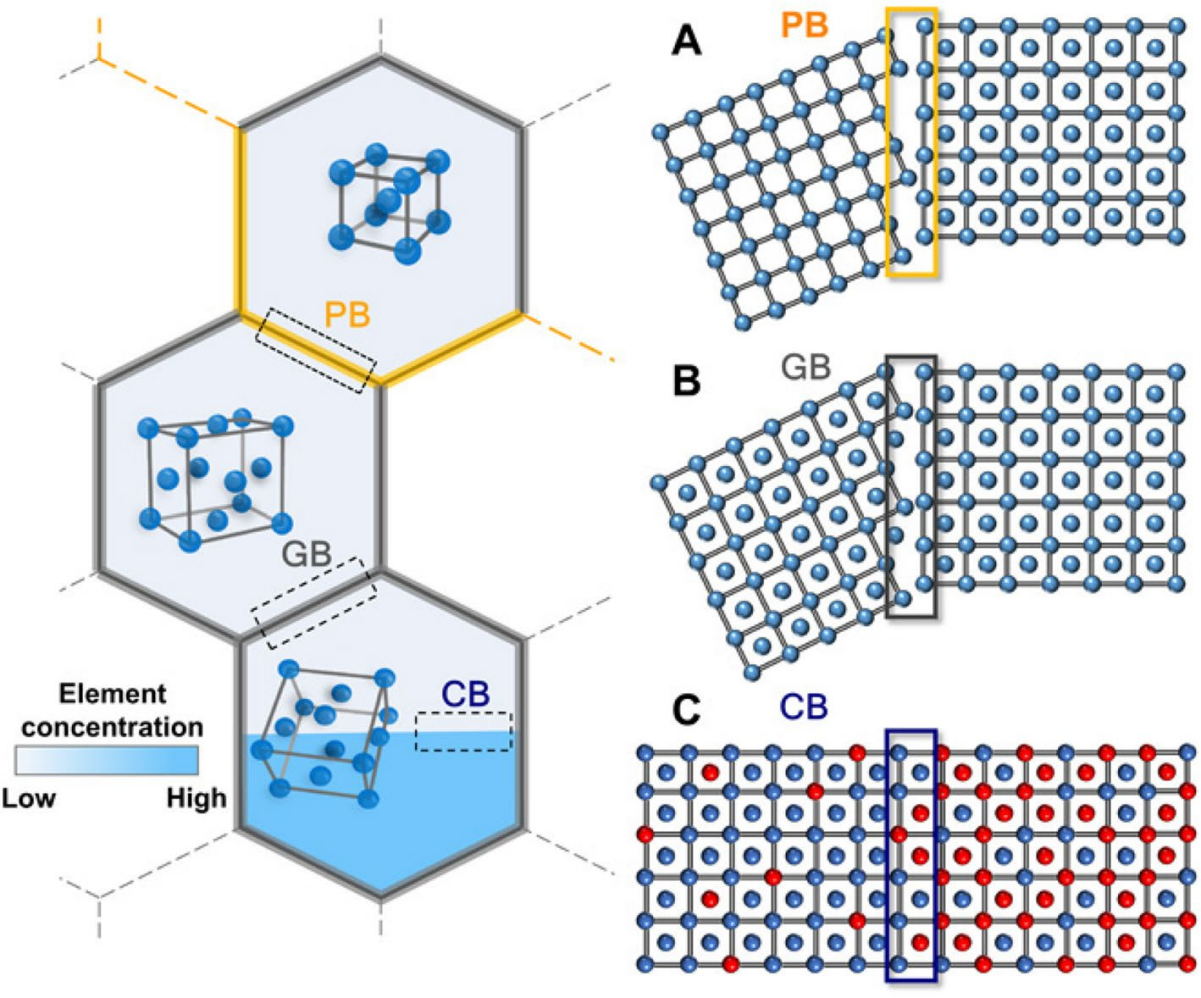
Schematic illustration of phase boundary (PB), Grain boundary (GB), and Chemical Boundary (CB) (Ding et al. 2020). All figures are reproduced with permission

Chemical heterogeneity through specific thermo-mechanical processing routes provides unique strengthening mechanisms that are not available in chemically homogeneous steels. The enhancement of strength in these materials is primarily attributed to the following two factors:

First, the formation of "chemical boundaries (CB)" acts as a physical barrier to dislocation motion. After full austenitization in Step 2, alloying elements remain heterogeneously distributed within individual austenite grains, giving rise to internal boundaries where the crystallographic orientation is identical, but the chemical composition differs. Although these boundaries are not as defined as conventional crystallographic grain boundaries, distinct regions with abrupt changes in chemical composition are clearly present; these are referred to as chemical boundaries. The differences among phase boundaries (PB), grain boundaries (GB), and chemical boundaries (CB) are schematically illustrated in Fig. 14. While GBs block phase propagation via crystallographic discontinuity, CBs prevent martensitic lath growth by reducing the local transformation driving force due to Mn enrichment. As a result, even without crystallographic orientation changes, CBs refine the martensite substructure. This structural refinement increases yield strength by providing potent barriers to both phase transformation propagation during quenching and dislocation motion during deformation (Ding et al 2020).

**Fig.15:**
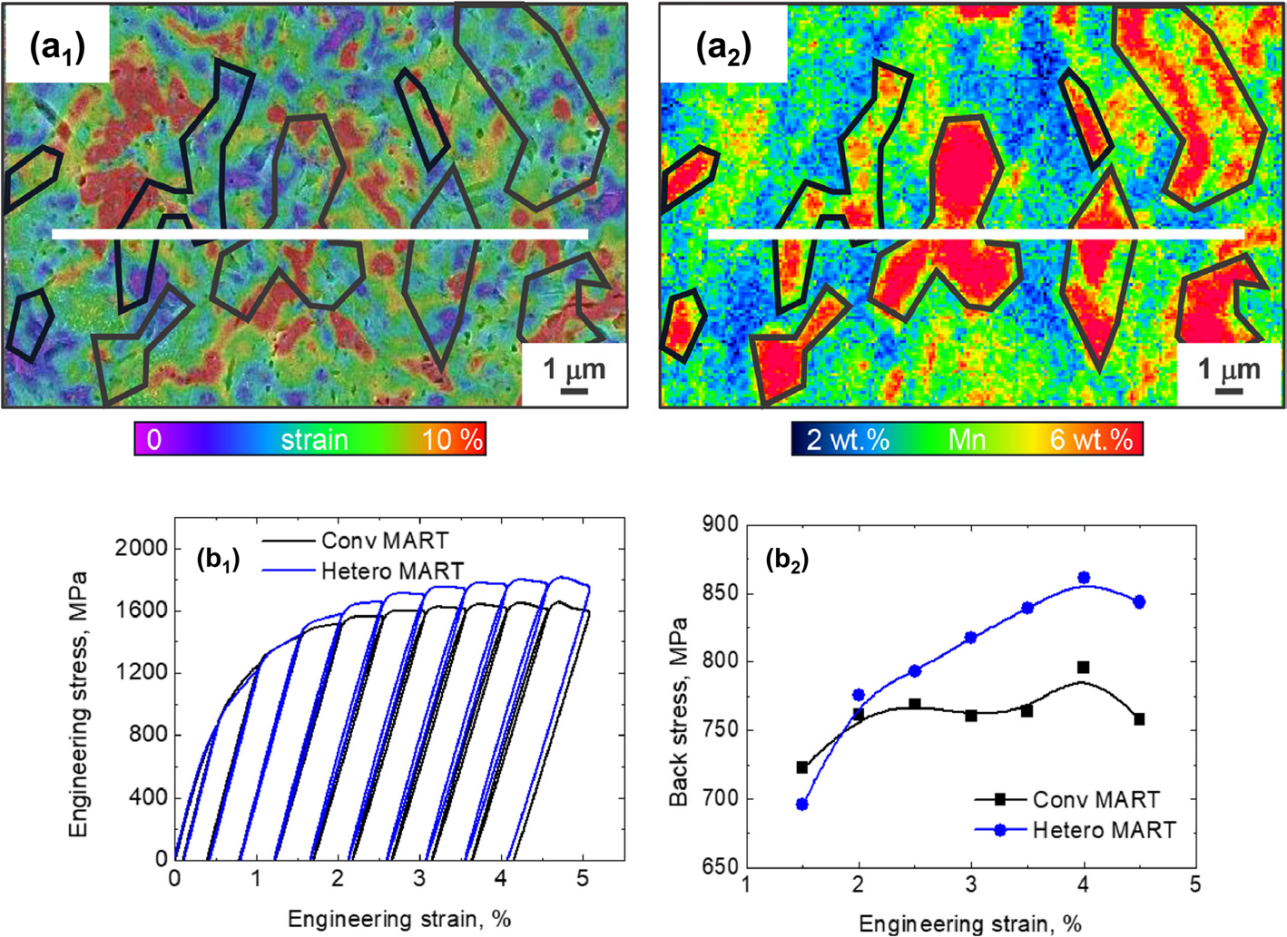
**a** Micro-strain distribution in chemically heterogeneous steel. **b** Comparison of back stress between chemically homogeneous and heterogeneous steel (Kim et al. 2022a). All figures are reproduced with permission

Second, the fluctuation of alloying elements induces strain partitioning, which leads to an enhancement of back stress. In chemically heterogeneous steels, the Mn-rich and Mn-poor regions exhibit different local yield strengths, creating a significant mechanical contrast. During deformation, this strength difference causes the harder (Mn-poor) and softer (Mn-rich) regions to deform at different rates, as evidenced by micro-strain distribution measurements where the Mn-rich regions show relatively lower micro-strain (Fig. 15a). This mechanical incompatibility promotes strain partitioning between the regions (Fig. 15b), resulting in the generation of geometrically necessary dislocations (GNDs) at the chemical boundaries, demonstrating that chemical boundaries impede dislocation motion (Kim et al. 2022a). Numerous studies have reported that this heterogeneity-driven back stress enhances the work-hardening rate, allowing the steel to achieve ultra-high tensile strength without a premature loss of load-bearing capacity (Gu et al. 2022; Liu et al. 2024a; Kaboli et al. 2026).

### Enhancement of ductility

**Fig. 16:**
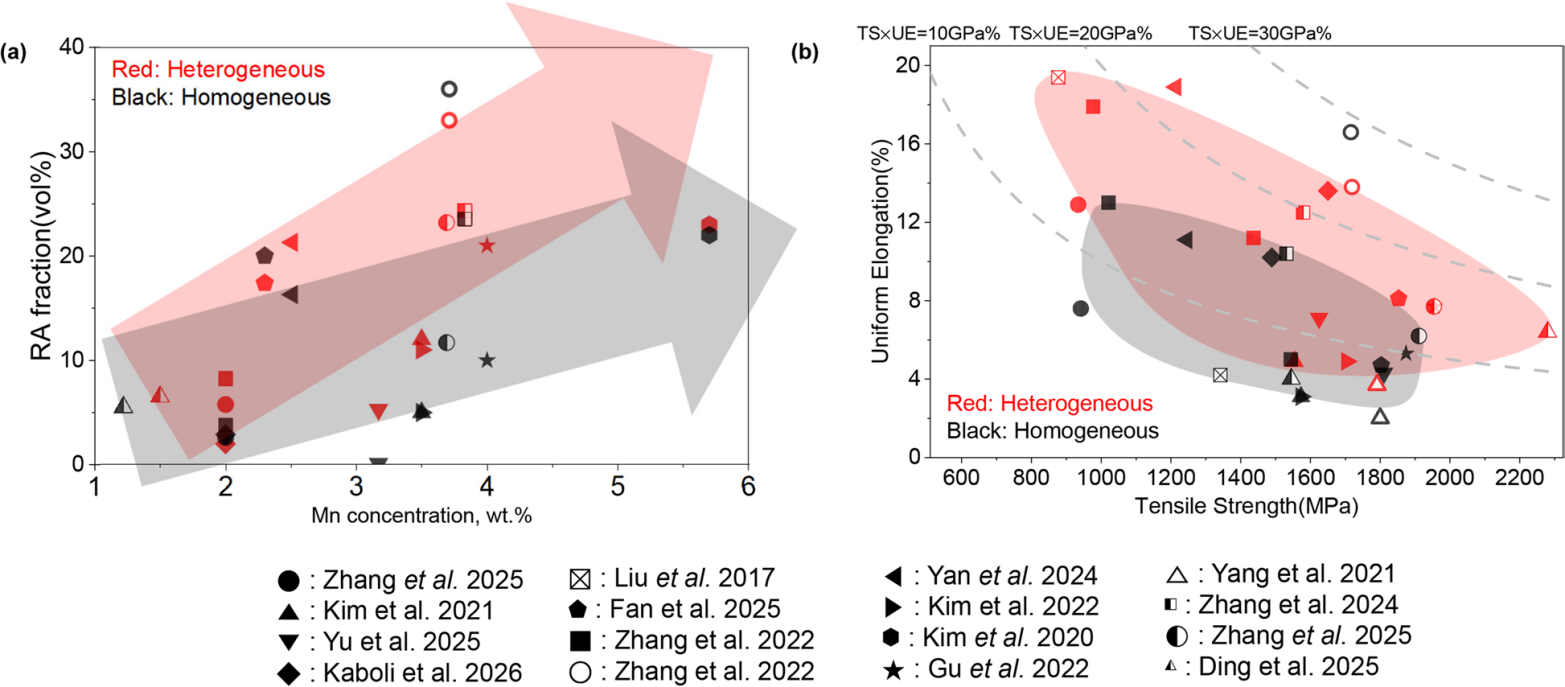
**a** Relationship between Mn concentration and retained austenite(RA) fraction in chemically heterogeneous steels. **b** Comparison of tensile strength and uniform elongation relation between chemically heterogeneous and chemically homogeneous steels

**Fig. 17:**
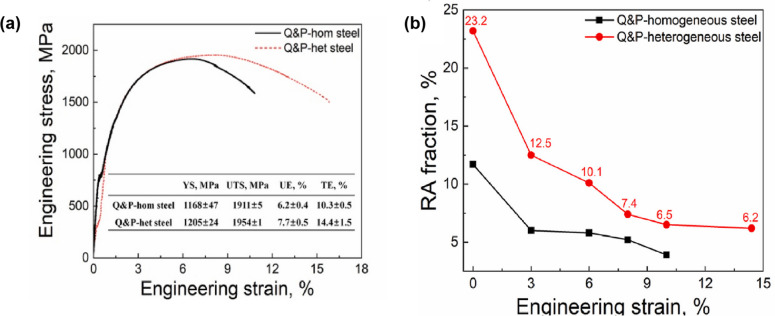
Improvement in mechanical properties of a 0.39C–3.69Mn steel due to an increased austenite fraction. **a** Engineering stress–strain curves and the corresponding tensile properties. **b** Evolution of the RA fraction as a function of engineering strain. (Yang et al. 2025). All figures are reproduced with permission

The superior ductility of chemically heterogeneous steels is primarily driven by the enhanced stabilization of retained austenite (RA) and its unique transformation behavior. The chemical heterogeneity strategy significantly increases the RA fraction and its mechanical stability. By localizing Mn and C within specific regions before full austenitization, RA is preferentially stabilized in Mn-enriched areas. This method can achieve a higher RA fraction compared to homogeneous steels with the same bulk composition (Fig. 16a). For instance, using an initial pearlitic microstructure to concentrate alloying elements has been reported to double the RA fraction from 11.7% to 23.2% (Yang et al. 2024a) (Fig. 17).Similarly, in press-hardening steels (PHS), controlling the size and Mn/Cr enrichment of initial cementite particles increased stable RA fraction from 3.0% to 6.5% (Ding et al. 2025). This increased RA fraction leads to an excellent combination of ductility and ultra-high strength (Fig. 16b). Furthermore, the concentration gradient creates stable spectrum of austenite, leading to a sustained TRIP effect. Unlike homogeneous steels where RA may transform all at once, the fluctuating Mn concentration in heterogeneous steels results in a broad distribution of austenite stability. During deformation, this gradient ensures that the transformation-induced plasticity (TRIP) effect occurs progressively and continuously over a wide strain range. This sustained work-hardening effectively delays the onset of necking, enabling an excellent combination of ultra-high strength and high total elongation—such as 2 GPa strength with 7.7%–11.2% elongation (Liu et al. 2021; Ding et al. 2025).

### Improved resistance to extreme environments

**Fig. 18:**
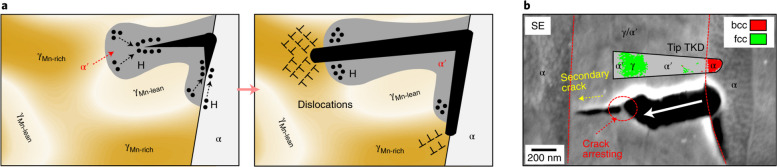
**a** Schematic illustration of crack propagation behavior and the corresponding crack-arresting mechanism in chemically heterogeneous steel. **b** Experimental observation of crack arresting at the chemical boundary in a chemically heterogeneous medium-Mn steel (Sun et al. 2021). All figures are reproduced with permission

Chemically heterogeneous steels also exhibit enhanced performance in extreme conditions, particularly regarding hydrogen embrittlement (HE) resistance, which is a critical challenge for high-strength alloys. The chemical boundaries and heterogeneous microstructure act as effective hydrogen traps and crack-arresting sites. Traditional strategies rely on grain refinement or precipitation to mitigate HE. In contrast, chemically heterogeneous steels utilize chemical boundaries (CBs) to arrest hydrogen-induced crack propagation. It has been observed that cracks initiated in hydrogen-charged environments are effectively deflected or stopped when they encounter a CB (Fig. 18). The high fraction of stable retained austenite (RA) in Mn-rich regions plays a critical role in preventing hydrogen embrittlement. Austenite acts as a hydrogen trap because it has a much higher solubility and lower diffusivity for hydrogen compared to martensite, effectively sequestering hydrogen and blocking its migration to crack tips. Furthermore, these Mn-rich zones have a low phase-transformation driving force, allowing them to remain as stable austenite rather than transforming into brittle martensite during deformation. This leads to a "crack-blunting" effect that physically stops crack growth. This heterogeneity-based strategy has been proven to improve hydrogen resistance by more than twofold compared to conventional methods like grain refinement (Sun et al. 2021).

## Future perspectives and challenges of chemically heterogeneous steel

### Microstructural control and mechanical property through utilization of chemical boundaries

One of the most distinctive microstructural features of chemically heterogeneous steels is the formation of chemical boundaries. As discussed above, chemical boundaries have been reported to play a role analogous to that of conventional grain boundaries. This analogy implies that chemical boundaries, like grain boundaries, can be actively exploited as an effective tool for microstructural control.

For example, one of the most widely used approaches for grain refinement in steels is the refinement of the parent phase, i.e. the prior austenite grain size, which directly leads to refinement of the final microstructure (Morito et al. 2005; Hidalgo & Santofimia 2016; Kim et al. 2019). To achieve this, various strategies have been employed, including cyclic heat treatments, grain-boundary pinning using precipitates stable at high temperatures, and severe deformation to refine austenite grains. In contrast, by exploiting chemical heterogeneity, it may be possible to refine transformation products even when the prior austenite grains are relatively coarse, without relying on additional processing steps or precipitation-strengthening alloying elements. Therefore, systematic investigations into grain refinement strategies based on chemical boundaries represent a crucial research topic for microstructural control in chemically heterogeneous steels.

In addition, fundamental studies on the role of chemical boundaries are still required. Although several studies have reported that chemical boundaries can impede dislocation motion and suppress crack propagation, the extent to which chemical boundaries function analogously to conventional grain boundaries remains insufficiently understood. From the perspective of stress or slip incompatibility, chemical boundaries differ fundamentally from grain boundaries, as the crystallographic orientation across a chemical boundary is identical. Consequently, slip systems are continuous across the boundary, which would, in principle, facilitate dislocation transmission. However, the ease of dislocation motion across chemical boundaries is expected to depend strongly on the degree of lattice distortion induced by local variations in alloying element concentration.

Accordingly, to establish a fundamental understanding of deformation and strengthening mechanisms associated with chemical boundaries, detailed investigations of dislocation–boundary interactions are essential. Experimental approaches analogous to those used in classical grain-boundary studies—such as micropillar compression, bicrystal deformation experiments, and direct observation of dislocation behavior at boundaries using transmission electron microscopy (TEM)—should be systematically applied to chemical boundaries. Such studies are indispensable for developing a comprehensive understanding of chemically heterogeneous steels.

In addition to experimental efforts, the integration of advanced multiscale modeling is imperative for future progress. For instance, phase-field modeling should be employed to simulate complex morphological features and chemical concentration gradients during thermal processing. Furthermore, crystal plasticity finite element method (CPFEM) models are needed to clarify the fundamental physical principles underlying. Such computational approaches will be instrumental in reducing experimental trial-and-error and enabling the rapid, precise design of steels' mechanical properties.

### Extension to alloying elements beyond Mn

To date, most studies on chemically heterogeneous steels have focused on utilizing Mn heterogeneity, since Mn is one of the most widely used alloying elements in steels and can be readily introduced through austenite reversion or cementite precipitation. However, chemical heterogeneity is not limited to Mn, and a broader range of alloying elements can be deliberately exploited in the design of chemically heterogeneous steels.

Among them, Cr is a particularly promising candidate, as it preferentially enriches in carbides, enabling the introduction of Cr-based chemical heterogeneity. Although several studies have explored Cr heterogeneity, the extent of research remains limited compared to that on Mn heterogeneity. Expanding this approach could provide new opportunities for microstructural control in Cr-containing steels, including stainless steels, where Cr plays a critical role. (Belde et al. 2015, 2016; Liu et al. 2024a, 2024b; Wang et al. 2024a; Ding et al. 2025) Similarly, Ni can also be utilized as a chemically heterogeneous element. As an austenite-stabilizing element, Ni can be heterogeneously distributed through austenite reversion, offering potential strategies for microstructural control in high-Ni martensitic steels designed for low-temperature applications (Wang et al. 2024a).

Beyond substitutional alloying elements, further attention should be given to exploiting heterogeneity in interstitial elements. Although interstitial elements such as C diffuse rapidly and are often assumed to homogenize readily during high-temperature austenitization, their distribution can remain heterogeneous when substitutional elements that reduce the chemical potential of C—such as Mn—are heterogeneously distributed. In such cases, thermodynamic interactions between alloying elements inevitably lead to spatial variations in C concentration. Given that C, even in small amounts, exerts a profound influence on phase transformations and mechanical properties in steels, future studies should focus on microstructural design strategies and mechanical property optimization in chemically heterogeneous steels that explicitly consider the coupled heterogeneity of C and substitutional alloying elements.

### Extension to other metallic materials beyond steel

In steels, chemical heterogeneity is typically introduced through pre-annealing in a temperature range where multiple phases coexist prior to full austenitization, owing to differences in the solubility of alloying elements among the constituent phases. The reason chemical heterogeneity can be readily introduced through heat treatment in steel lies in their high-temperature phase transformation behavior. In general, ferrite with a body-centered cubic (BCC) structure is stable at low temperatures, whereas austenite with a face-centered cubic (FCC) structure is stable at high temperatures. This inherent phase stability inevitably gives rise to a multiphase region in which the BCC and FCC phases coexist.

Accordingly, the concept of chemical heterogeneity is not limited to steel but can, in principle, be applied to all metallic materials that undergo high-temperature phase transformations. A representative example is titanium. In Ti alloys, the hexagonal close-packed (HCP) α phase is stable at room temperature, while the body-centered cubic β phase becomes stable at elevated temperatures. By conducting heat treatment within the α + β two-phase region to introduce chemical heterogeneity, followed by rapid heating to obtain a single β phase, chemical heterogeneity can be retained within the β phase. Indeed, only a limited number of studies have reported improvements in mechanical properties in Ti alloys by exploiting chemical heterogeneity, highlighting the need for further systematic investigations and demonstrating the potential for extending the chemical heterogeneity concept beyond steels to other alloy systems (Wu et al. 2023; Jia et al. 2024).

### Adjusting to industry processing line and evaluating various in-service properties

Chemically heterogeneous steels have emerged relatively recently as a novel microstructural control strategy, and most studies to date have focused on microstructural evolution as a function of processing conditions (Steps 1, 2, and 3 heat treatments) and the resulting mechanical properties, particularly tensile properties. However, many reported studies rely on processing conditions that are difficult to implement in practical steel manufacturing routes, such as applying pre-annealing treatments for several hours in Step 1 to maximize chemical heterogeneity or employing flash heating during Step 2. Consequently, there is a clear need for process-friendly studies aimed at optimizing the introduction of chemical heterogeneity under industrially relevant conditions, such as those encountered in continuous annealing lines.

Furthermore, while current microstructural designs primarily rely on static heat treatments to introduce chemical heterogeneity, integrating mechanical deformation—such as rolling processes in industrial lines—presents a promising strategy to further maximize these compositional fluctuations. The fundamental reason is that the accumulation of strain energy during deformation alters the local thermodynamic equilibrium, providing an additional mechanical driving force. This synergy of thermal and mechanical driving forces pronounced nanoscale elemental partitioning (Cheng et al. 2020).

With respect to mechanical properties, most existing studies have primarily evaluated tensile behavior. However, for chemically heterogeneous steels to be utilized as structural materials, investigations must extend beyond tensile properties to include a broader range of in-service properties. Representative examples include formability-related properties such as bendability and stretch-flangeability, as well hydrogen embrittlement resistance, which are critical for practical structural applications. Likewise important is the comprehensive evaluation of fracture and impact toughness, which governs the structural reliability of these materials under dynamic loading but currently lack sufficient experimental data. Therefore, systematic studies are required to elucidate how chemical heterogeneity and chemical boundaries influence these in-service properties.

## Summary

**Fig. 19:**
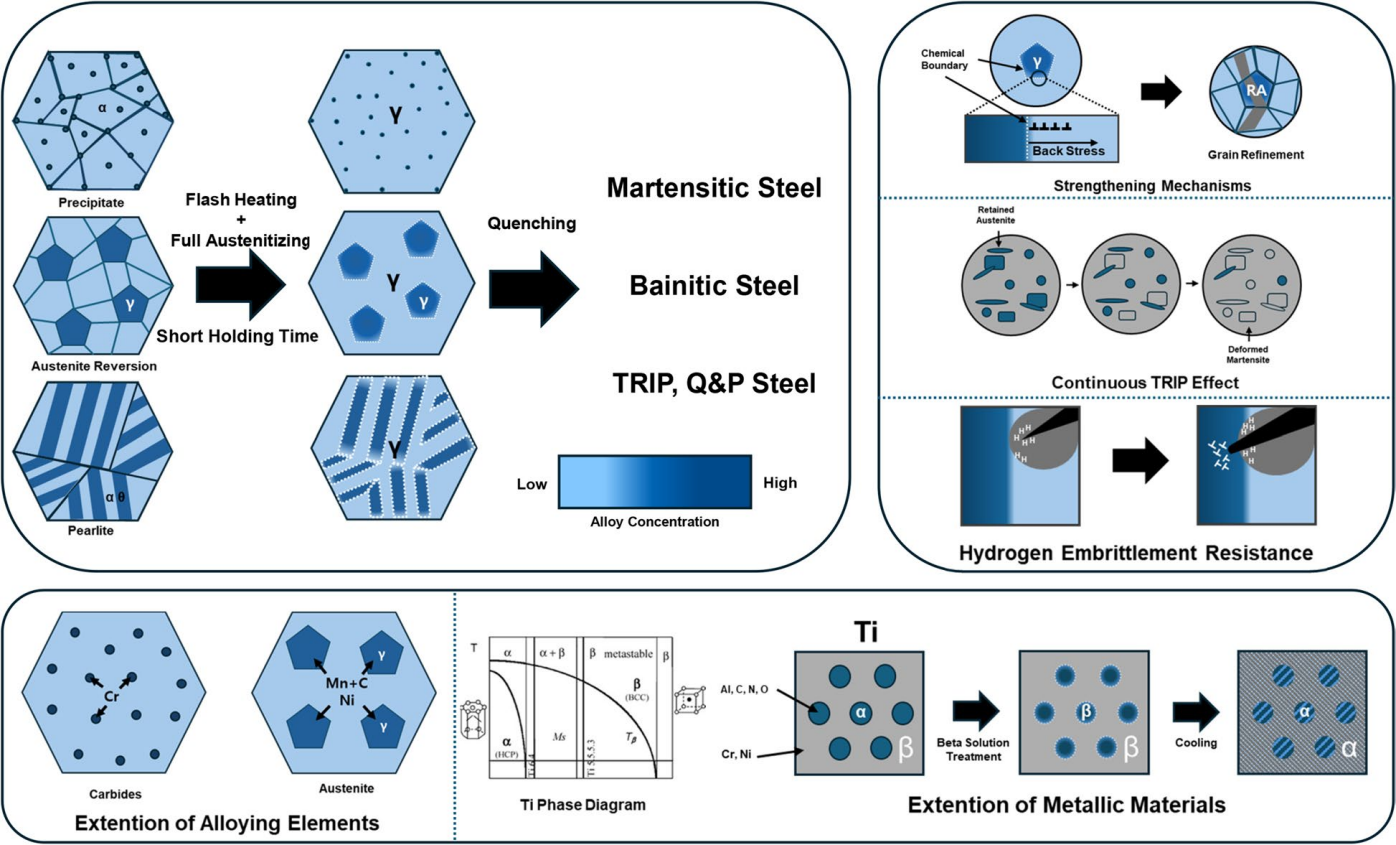
Summary of the processing routes, strengthening mechanisms, and future perspectives for chemically heterogeneous steels. Ti phase diagram is adapted from (Arrazola et al. 2009)

Chemically heterogeneous steels represent a novel microstructural design strategy that overcomes the limitations of conventional homogeneous steels by intentionally introducing heterogeneous chemical distributions. This approach involves (i) generating chemical heterogeneity in multiphase regions, (ii) retaining it during austenitization via controlled heating and diffusion kinetics, and (iii) applying subsequent heat treatments to produce diverse microstructures such as martensitic, bainitic, and Q&P steels. As a result, heterogeneous austenite exhibits distinct phase transformation characteristics, including multi-step martensitic transformation, modified bainitic kinetics, and enhanced retained austenite stability. These features lead to superior mechanical performance through chemical boundary strengthening, strain partitioning–induced back stress, and a sustained TRIP effect, along with improved resistance to extreme environments such as hydrogen embrittlement. Since this concept has been relatively recently introduced in AHSSs, most studies have primarily focused on tensile properties. Therefore, other mechanical properties such as impact toughness, stretch-flangeability, and formability should be systematically investigated to clarify the influence of chemical heterogeneity. For further extension of this concept, not only Mn but also other alloying elements such as Cr and Ni should be explored. In addition, this concept can be extended to other metallic materials that undergo high-temperature phase transformations, such as Ti alloys (Fig. 19).

## Data Availability

Most of the data was adopted from previously published works with permission from the publishers. Additional private datasets generated or used during the current study are available from the corresponding author upon reasonable request.
